# Importance of endoscopic and histological evaluation in the management of immune checkpoint inhibitor-induced colitis

**DOI:** 10.1186/s40425-018-0411-1

**Published:** 2018-09-25

**Authors:** Hamzah Abu-Sbeih, Faisal S. Ali, Wenyi Luo, Wei Qiao, Gottumukkala S. Raju, Yinghong Wang

**Affiliations:** 1 Department of Gastroenterology, Hepatology and Nutrition, The University of Texas MD Anderson Cancer Center, 1515 Holcombe Blvd., Unit 1466, Houston, TX 77030 USA; 2 Department of Pathology/Laboratory Medicine, The University of Texas MD Anderson Cancer Center, Houston, TX USA; 3 Department of Biostatistics, The University of Texas MD Anderson Cancer Center, Houston, TX USA

**Keywords:** Immune-checkpoint inhibitors, Colitis, Endoscopy, Histology, Diarrhea

## Abstract

**Background:**

Immune checkpoint inhibitors (ICPI) are efficacious treatments for advanced malignancies but can result in immune mediated diarrhea and colitis (IDC). Currently, the guidelines for the treatment of IDC depend only on clinical symptoms. Endoscopic and histologic features of such adverse events are not well studied in a manner that can help to gauge treatment plans. We aimed to characterize endoscopic and histologic features of IDC and to assess their association with clinical outcomes.

**Methods:**

Our study included patients who had undergone endoscopy for IDC (1/2010 to 3/2018). Patients with GI infection at time of onset were excluded. High-risk endoscopic features were ulcers deeper than 2 mm, larger than 1 cm, and extensive colonic involvement. Univariate and multivariate logistic regression were performed to assess the association of endoscopic and histological features with clinical outcomes.

**Results:**

A total of 182 patients was included; most were white (92%), males (65%) with a mean age of 60 years. Median time from ICPI initiation to IDC was 7 weeks. Fifty-three percent had grade 3–4 diarrhea, and 32% grade 3–4 colitis. Forty-nine patients had mucosal ulcerations, 66 non-ulcerative inflammation and 67 normal endoscopy. Calprotectin was higher in patients with ulceration (*P* = 0.04). The sensitivity of lactoferrin to detect histologic and endoscopic inflammation was 90% and 70% respectively. Patients who underwent endoscopy earlier than 7 days after IDC onset had shorter duration of IDC symptoms and duration of steroid treatment than those who underwent endoscopy after 7 days of IDC onset (*P* = 0.026 and *P* = 0.053, respectively). Patients who underwent endoscopy > 30 days of symptom onset required longer duration of steroids (*P* = 0.02), had more recurrent symptoms (*P* < 0.01) and received later infliximab/vedolizumab add-on therapy than did those who underwent endoscopy ≤30 days (*P* = 0.03). High-risk features were associated with more frequent (*P* = 0.03) and longer duration (*P* = 0.02) hospitalization and infliximab/vedolizumab requirement (*P* < 0.01). Patients with active histological inflammation had more recurrence (*P* < 0.01) and repeat endoscopy (*P* < 0.01). Repeat endoscopy was required in 47 patients. A multivariate logistic regression revealed that longer ICPI treatment was associated with more frequent hospitalizations (OR 1.00; 95%CI 1.00–1.01; *P* < 0.01) and high-risk endoscopic features were associated with the requirement of infliximab/vedolizumab (OR 3.89; 95%CI 1.68–9.01; *P* < 0.01).

**Conclusion:**

High risk endoscopic features and active histologic inflammation represent important markers of disease severity with clinical implications and should be used in a timely manner to devise IDC-focused treatment algorithms.

**Electronic supplementary material:**

The online version of this article (10.1186/s40425-018-0411-1) contains supplementary material, which is available to authorized users.

## Background

Immune checkpoint inhibitors (ICPIs) represent an efficacious cancer treatment that improves survival in metastatic malignancies [1, 2]. Initially, inhibitors of cytotoxic T-lymphocyte antigen-4 (CTLA-4), followed by inhibitors of the programmed cell death receptor-1 (PD-1) and PD-ligand 1 (PD-L1), showed efficacy and improved survival in patients with melanoma [1–3]. Subsequently, ICPIs demonstrated high effectiveness in the treatment of non-melanoma solid tumors as well, such as non-small cell lung cancer and renal cell carcinoma [4, 5]. Boasting such a positive profile, the indications for ICPI therapy are expected to increase in the near future. Thus, it is critical to be well versed in the adverse effects of these drugs and their optimal management strategies.

Owing to the immunological mechanism of ICPIs, their adverse effects collectively fall under the umbrella of immune-related adverse events (irAEs). Although ICPI-induced irAEs can affect virtually any organ system, those affecting the gastrointestinal (GI) tract are among the most common severe irAEs that lead to ICPI treatment discontinuation [6–8]. Wang et al. reported in their meta-analysis that the incidence of grade 3 and 4 colitis was 9.1% with CTLA-4 monotherapy, 1.3% with PD-1/L1 therapy, and 13.6% with combination therapy [9]. GI-irAEs that affect the lower GI tract present as diarrhea, alone or with additional signs, symptoms, and diagnostic characteristics of colitis. Preliminary evidence on GI-irAEs sheds light on the endoscopic and histological characterization of this entity [10–12]. Additionally, a significant overlap has been found between the endoscopic and histological profile of GI-irAEs and that of inflammatory bowel disease (IBD), where it is used to guide IBD mangement [13]. Therefore, it is necessary to characterize the endoscopic and histological features of GI-irAEs to guide management decisions.

In regards to GI-irAE treatment, the American Society of Clinical Oncology and Society for Immunotherapy of Cancer provide their recommendations based on the Common Terminology Criteria for Adverse Events (CTCAE) grading system [6, 14]; grade 2 irAEs should prompt the initiation of corticosteroid treatment, while, grade 3 and higher indicate the need for hospitalization and consideration of a non-corticosteroid drug such as infliximab. The caveat with current recommendations is their dependence on clinical symptoms only, which may not be the most accurate measure of disease severity in all cases. Also noteworthy is that these guidelines are based on very low level evidence, which consists mainly of expert consensus and very few studies. In addition, the current recommendations do not identify surrogate markers that can aid in prompting additional non-corticosteroid therapy. Data on the appropriate and timely administration of an add-on immunosuppressive drugs such as infliximab or vedolizumab, a monoclonal antibody that has shown efficacy in the treatment of GI-irAE [15], are lacking. Endoscopic and histological findings could provide useful information to help fill the above-mentioned knowledge gaps.

We previously reported a study of 53 patients in which we characterized the endoscopic and histological features of ICPI-induced diarrhea and colitis (IDC) [10]; we found that the presence of ulceration on endoscopy was a surrogate marker for steroid-refractory IDC. However, the above-mentioned knowledge gaps, especially the lack of characterization, which affects disease course and could prompt management decisions, were left unfilled. The aim of this study was to characterize the endoscopic and histological features of IDC and assess their association with clinical characteristics and outcomes to improve upon the currently available guidelines and provide an IDC focused treatment algorithm.

## Methods

### Patient selection and data collection

This was a retrospective study of patients who received ICPI treatment and underwent endoscopic and histological evaluation for IDC at The University of Texas MD Anderson Cancer Center between January 2010 and March 2018. Approval for this study was obtained from the Institutional Review Board at MD Anderson. Informed consent was waived. We included adult patients who (1) had received ICPI; (2) had developed IDC; and (3) had undergone endoscopy with tissue collection. IDC diagnosis was established according to the treating gastroenterologist or oncologist based on clinical, endoscopic and/or histological characteristics. Patients whose diarrhea was attributed to other etiologies were excluded. We collected data regarding patients’ characteristics, medical and oncological history, IDC, computed tomography (CT) imaging, endoscopic findings, histological features, and clinical outcomes. We collected cancer stage only for patients with melanoma and solid tumors, as defined by the American Joint Committee on Cancer Staging System, 7th edition.

### Clinical evaluation of IDC

#### Clinical characteristics

The highest grade of IDC, as reported in the medical chart by the treating physician using CTCAE, 4.03, was recorded. Symptom duration was measured from the time of symptom onset to resolution. In addition, we recorded the time from ICPI initiation to IDC onset. Immunosuppressant agents used for IDC treatment included steroids, infliximab, and vedolizumab. The duration of corticosteroid therapy was reported as the cumulative time on corticosteroids.

#### Endoscopic evaluation

Data relating to endoscopy included endoscopy type, gross description, and colitis distribution. The time from the onset of IDC to the first endoscopic evaluation was recorded and was categorized as > 30 days or ≤ 30 days. Gross description on endoscopy was characterized as the presence of mucosal ulcerations, non-ulcerative inflammation (erythema, exudate, loss of vascular pattern, edema, friability, and erosions), or normal appearance. Based on the clinical experience of the primary investigators of this study and the established endoscopic scoring criteria for IBD, endoscopic features were categorized retrospectively as low- or high-risk to indicate the likelihood of IDC to be refractory to steroids. High-risk features included either high-risk ulcers, as reported by the endoscopist, (deeper than 2 mm and/or larger than 1 cm in surface area) or extensive colitis (endoscopic inflammation involving the colon proximal to the splenic flexure). The distribution of colitis was classified as terminal ileum with or without colon, left colon only, right colon only, left and right colon, or none. Of note, the term extensive colitis includes the involvement of the terminal ileum with the colon or the involvement of the right and left colon. Endoscopic presentation of IDC was categorized into Crohn’s colitis (CC)- or ulcerative colitis (UC)-like based on its resemblance to IBD pattern.

#### Histological examination

Biopsies were obtained from both normal and abnormal areas of the left and right colon as well as the terminal ileum, depending on the extent of endoscopy. GI pathologists then reviewed the histopathological reports and slides. Active histological inflammation features included neutrophilic or eosinophilic infiltrate, cryptitis, crypt abscess, and apoptosis. Chronic inflammation features included basal lymphoplasmacytic infiltrate, cryptic architectural distortion, or Paneth cell metaplasia. The microscopic pattern of intraepithelial lymphocytosis was categorized as active inflammation.

#### Clinical outcomes

The primary clinical outcomes were the need for and length of hospitalization, intensive care unit (ICU) admission, the recurrence of IDC symptoms, the need for repeat endoscopy because of persistent or recurrent symptoms, and colonic perforation. As a secondary outcome, we measured the overall survival (OS) duration, which was defined as the time from ICPI initiation until death or last follow-up clinical encounter. Additionally, we reported the clinical remission rate, which was defined as complete sustained subsidence of IDC symptoms after tapering off steroids. Similarly, we reported the endoscopic and histological remission rates for patients who underwent repeat endoscopy. Endoscopic remission was defined as the resolution of inflammation or healing of mucosal ulceration. Histological remission was defined as the absence of active histological features.

### Statistical analysis

Statistical analyses were carried out using SAS version 9.4 (SAS Institute, Cary, NC) and SPSS version 24.0 (SPSS, Inc., Chicago, IL). The distribution of continuous variables was summarized using the mean, median, standard deviation (SD), and range. The distribution of categorical variables was summarized by frequencies and percentages. Continuous variables were compared between subgroups using the Wilcoxon rank-sum test or Kruskal-Wallis test (for more than two groups). Fisher exact test or chi-square test was used to evaluate associations between categorical variables. Univariable and multivariable logistic regression analysis were conducted to assess the association between clinical factors and outcomes. Kaplan-Meier curves were used to estimate unadjusted OS. Log-rank tests were used to compare OS between groups. All statistical tests were 2-sided. All statistical tests were two-sided. *P* values < 0.05 were considered statistically significant.

## Results

### Patient characteristics

**Fig. 1:**
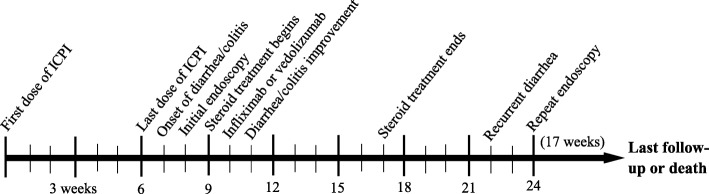
Timeline of events in relation to ICPI-induced colitis, based on the median number of weeks

This study included 182 patients who underwent endoscopic and histological evaluation for IDC. A schema of our patient population is shown in Additional file 1: Figure S1. One hundred sixty-seven (91.8%) were white, and 119 (65.4%) were males. The mean age was 60 years (Table 1). Melanoma was the most common malignancy in 77 (42.3%) patients. Concerning ICPI therapy, 71 (39.0%) had received CTLA-4 inhibitors, 67 (36.8%) PD-1/L1 inhibitors, and 44 (24.2%) combination therapy. The median time from ICPI initiation to IDC onset was 7 weeks (interquartile range, 1–35 weeks). Eighty-six (47.3%) patients had grade 2 and 59 (32.4%) had ≥ grade 3 colitis. Abnormal CT findings suggestive of colitis were detected in 43 (38.1%) of the 113 patients who underwent CT imaging. The mean duration of GI symptoms was 1 month (SD, 2 months). A timeline of events of our study is shown in Fig. 1.

**Table 1:** Association between patient characteristics and treatment group

Characteristic	Immunosuppressant *N* = 141	No immunosuppressant *N* = 41	*P* value
Age in years, mean (SD)	60 (15)	58 (19)	0.371
Male sex, n (%)	94 (66.7)	25 (61.0)	0.576
White race, n (%)	135 (95.7)	32 (78.0)	0.001
Comorbidities present, n (%)	79 (56.0)	28 (68.3)	0.207
Smoking, n (%)	74 (52.5)	22 (53.7)	1.000
NSAID, n (%)	76 (53.9)	20 (48.8)	0.597
Malignancy type, n (%)			0.016
Melanoma	62 (44.0)	15 (36.6)	
Solid	74 (52.5)	19 (46.3)	
Hematological	5 (3.5)	7 (17.1)	
Cancer stage^a^, n (%)			1.000
III	13 (9.6)	3 (8.8)	
IV	122 (90.4)	31 (91.2)	
Checkpoint inhibitor type, n (%)			0.051
CTLA-4	59 (41.8)	12 (29.3)	
PD-1/L-1	45 (31.9)	22 (53.7)	
Combination^b^	37 (26.2)	7 (17.1)	
Diarrhea grade			< 0.001
1	4 (2.8)	20 (48.8)	
2	43 (30.5)	17 (41.5)	
3–4	94 (66.7)	4 (9.8)	
Colitis grade			< 0.001
1	23 (16.3)	14 (34.1)	
2	59 (41.8)	27 (65.9)	
3–4	59 (41.8)	0 (0.0)	
Endoscopic evaluation			0.223
Flexible sigmoidoscopy	33 (23.4)	14 (34.1)	
Colonoscopy	108 (76.6)	27 (65.9)	
Distribution of colitis			< 0.001
Terminal ileum involved	10 (7.1)	1 (2.4)	
Right colon only	4 (2.8)	1 (2.4)	
Left colon only	48 (34.0)	9 (22.0)	
Entire colon	39 (27.7)	3 (7.3)	
Normal	40 (28.4)	27 (65.9)	
IBD like endoscopic pattern^c^			0.229
Crohn’s colitis	32 (31.7)	7 (50.0)	
Ulcerative colitis	69 (68.3)	7 (50.0)	

Abbreviation: *NSAID*, non-steroidal antiinflammatory drugs, *CTLA-4* cytotoxic T-lymphocyte antigen-4, *PD-1/L-1* programmed cell death receptor-1 and ligand 1, *SD* standard deviation

^a^ American Joint Committee on Cancer (AJCC) Cancer Staging System, 13 patients are missing

^b^ Combination: ipilimumab + nivolumab

^c^ Only 115 patients were included for the IBD like endoscopic pattern evaluation

### IDC treatment

One hundred forty-one (77.5%) patients required immunosuppressant treatment, whereas 41 (22.5%) received symptomatic management only. The mean duration of corticosteroid therapy was 2 months (SD, 2 months). Among patients who received immunosuppressive therapy, 47 (25.8%) received mesalamine. A total of 53 (29.1%) patients required the addition of infliximab or vedolizumab to steroid therapy; 42 for the initial episode of diarrhea, whereas 11 for recurrent disease.

### Endoscopic evaluation

#### Distribution and endoscopic findings

Colitis involved the entire colon in 42 (23.1%) patients, and in 11 patients (6.0%), there was evidence of ileal involvement. In 57 (31.3%) patients, colitis was limited to the left colon, whereas in 5 (2.7%), it was limited to the right colon. In regards to the endoscopic features of colitis, 49 (26.9%) patients had ulcers, 66 (36.3%) had non-ulcerative inflammation and 67 (36.8%) had normal endoscopy (Additional file 1: Table S1). High-risk endoscopic features were observed in 71 (39.0%) patients (Additional file 1: Figure S2). Among patients with abnormal endoscopic findings, 39/115 (33.9%) patients had CC-like presentation and 76/115 (66.1%) had UC-like presentation. No significant differences regarding clinical characteristics and outcomes were observed between patients who had CC-like disease and those who had UC-like disease (Table 2). The endoscopic and histopathological features of IDC are demonstrated in Additional file 1: Figures S3 and S4.

**Table 2:** Clinical characteristics and outcomes according to IBD-like endoscopic pattern

Characteristic	Crohn’s colitis *N* = 39	Ulcerative colitis *N* = 76	*P* value
Duration of symptoms (days, SD)			
IV steroids, n (%)	19 (61.3)	49 (75.4)	0.229
Infliximab/vedolizumab, n (%)	12 (37.5)	29 (42.0)	0.828
Duration from onset to recurrence (days, SD)	159 (158)	112 (118)	0.358
Duration of steroid (days, SD)	70 (117)	60 (43)	0.529
Diarrhea grade, n (%)			0.118
1	5 (12.8)	5 (6.6)	
2	145 (35.9)	18 (23.7)	
3–4	20 (51.3)	53 (69.7)	
Colitis grade, n (%)			0.201
1	7 (17.9)	5 (6.6)	
2	16 (41.0)	36 (47.4)	
3–4	16 (41.0)	35 (46.1)	
High-risk endoscopic features	23 (59.0)	48 (63.2)	0.689
Active histologic inflammation	33 (84.6)	67 (88.2)	0.574
Outcomes, n (%)			
Hospitalization	30 (76.9)	65 (85.5)	0.301
Duration of hospitalization (days)	8 (6)	8 (7)	0.855
ICU admission	2 (5.1)	3 (3.9)	1.000
Recurrence	15 (38.5)	16 (21.1)	0.074
Repeat endoscopy	12 (30.8)	15 (19.7)	0.245

Abbreviation: *ICU* intensive care unit, *IV* intravenous, *SD* standard deviation

#### Endoscopy timing

The median time from IDC diagnosis to endoscopic evaluation was 7 days (SD, 52). Most patients (142; 78%) underwent endoscopic evaluation ≤30 days of IDC onset (Table 3). Patients who underwent endoscopy ≤30 days of IDC diagnosis had shorter duration of steroid treatment (*P* = 0.019) and less recurrence of symptoms (*P* = 0.001). Additionally, although statistically insignificant, they had shorter duration of symptoms (*P* = 0.062), required less IV steroids (*P* = 0.054) and ICU admissions (*P* = 0.072). The initiation of infliximab/vedolizumab therapy in patients who underwent endoscopy > 30 days after IDC onset was later than in those who underwent endoscopy ≤30 days (*P* = 0.030).

**Table 3:** Clinical outcomes of patients according to the timing of endoscopy from IDC onset

Characteristic	Endoscopy > 30 days of onset *N* = 40	Endoscopy ≤ 30 days of onset *N* = 142	*P* value	Endoscopy > 7 days of onset *N* = 89	Endoscopy ≤ 7 days of onset *N* = 93	*P* value
IV steroids, n (%)	23 (57.5)	60 (42.3)	0.054	46 (66.7)	37 (56.9)	0.287
Duration of symptoms (days, SD)	54 (92)	26 (77)	0.062	47 (104)	19 (47)	0.026
Duration of steroid (days, SD)	87 (120)	53 (41)	0.019	74 (90)	49 (43)	0.053
Infliximab/vedolizumab, n (%)	8 (22.9)	34 (32.1)	0.395	26 (29.2)	27 (29.0)	1.000
Duration from onset to first infliximab/vedolizumab dose (days, SD)	31 (23)	15 (14)	0.030	23 (17)	14 (17)	0.154
Colonoscopy findings, n (%)			0.161			0.263
Ulcer	9 (22.5)	40 (28.2)		27 (30.3)	22 (23.7)	
Non-ulcerative inflammation	11 (27.5)	55 (38.7)		27 (30.3)	39,941.9)	
Normal	20 (50.0)	47 (33.1)		35 (39.3)	32 (34.4)	
High-risk endoscopic features, n (%)	14 (35.0)	57 (40.1)	0.587	37 (41.6)	34 (36.6)	0.544
Active histological inflammation, n (%)	29 (72.5)	100 (70.4)	0.847	63 (70.8)	66 (71.0)	1.000
Outcomes, n (%)						
Hospitalization	27 (67.5)	105 (73.9)	0.428	58 (65.2)	74 (79.6)	0.032
Duration of hospitalization (days, SD)	9 (7)	7 (6)	0.138	9 (7)	6 (7)	0.068
ICU admission	4 (10)	3 (2.1)	0.072	4 (4.5)	3 (3.2)	0.856
Recurrence	20 (50.0)	31 (21.8)	0.001	60 (67.4)	71 (76.3)	0.191

Abbreviation: *ICU* intensive care unit, *IV* intravenous, *SD* standard deviation

Patients who underwent endoscopy earlier than 7 days after IDC onset had shorter duration of IDC symptoms than those who underwent endoscopy after 7 days of IDC onset (*P* = 0.026; Table 3). Likewise, Patients who had endoscopy ≤ 7 days following IDC onset received shorter duration of steroid treatment than the other group (*P* = 0.053). Patients that had endoscopic evaluation within 1 week were admitted to the hospital more often than patients who had endoscopic evaluation after 1 week (*P* = 0.032). Although insignificant, the duration of hospitalization was longer in patients who had endoscopy after 7 days of IDC onset than in patients who had endoscopy within 7 days (*P* = 0.68).

#### Diagnostic laboratory results

Fecal lactoferrin was measured in 71 patients; positive in 60 (84.5%), of whom, 17/60 (29.3%) had ulcerative and 25 (41.7%) had non-ulcerative inflammation. Of the patients who had a positive lactoferrin assay, 54/60 (90.0%) had abnormal histological findings. For patients who had fecal lactoferrin tested, the sensitivity at detecting endoscopic inflammation was 70%, whereas 90% at detecting histological inflammation (Additional file 1: Table S2). Fecal calprotectin was measured for 39 patients, among them 17/39 (43.6%) had values <150mcg/g of stool. Calprotectin > 150mcg/g of stool had a sensitivity of 68% to detect abnormal endoscopic features, and 86% to detect histological active inflammation. The mean fecal calprotectin value was 465mcg/g of stool (SD, 363) in patients with ulceration, whereas in patients with normal endoscopic features, it was 152mcg/g of stool (SD, 133).

#### Characteristics of patients with grade 2 diarrhea (*n* = 60)

Patients who underwent endoscopy within 7 days of IDC onset had subsequently a duration of symptoms and hospitalization that are shorter than in patients who had endoscopy after 7 days of onset (*P* = 0.025 and *P* < 0.001, respectively; Additional file 1: Table S3). Endoscopic evaluation after 7 days of IDC onset was associated with higher rate of symptoms recurrence (*P* = 0.024) and lower rate of hospitalization than in timely endoscopy (*P* = 0.008). Patients who required immunosuppression for IDC had more frequently high-risk endoscopic features, active histological features, and symptoms recurrence (*P* = 0.063, *P* < 0.001, and *P* = 0.024, respectively; Additional file 1: Table S4).

#### Clinical characterization according to endoscopic and histological findings

Twenty-eight (15.4%) patients in our cohort had normal endoscopy and histology. These patients had lower grades of diarrhea (*P* = 0.010), less requirement for infliximab/vedolizumab therapy (*P* = 0.034), fewer hospitalizations (*P* = 0.021) and IDC recurrence (*P* = 0.037; Additional file 1: Table S5). The duration of symptoms and steroid treatment in patients with diarrhea and normal endoscopy and histology were 15 and 34 days, respectively, compared with 36 and 65 days, respectively, in all other patients. High-risk endoscopic features were associated with a higher frequency of infliximab or vedolizumab infusions (*P* < 0.001; Table 4) and more frequent (*P* = 0.028) and longer (*P* = 0.016) hospitalizations compared to non-high-risk features.

**Table 4:** Patients with endoscopic inflammation involvement

Characteristic	High-risk features^a^ *N* = 71	No high-risk features *N* = 111	*P* value
Duration of symptoms (days, SD)	41 (106)	27 (60)	0.301
IV steroids, n (%)	41 (66.1)	42 (58.3)	0.378
Infliximab/vedolizumab, n (%)	30 (46.2)	12 (15.8)	< 0.001
Mean duration from diagnosis to first recurrence (days, SD)	140 (147)	144 (121)	0.902
Outcomes, n (%)			
Hospitalization	58 (81.7)	74 (66.7)	0.028
Duration of hospitalization (days, SD)	9 (8)	6 (5)	0.016
ICU admission	3 (4.2)	4 (3.6)	0.656
Recurrence	20 (28.2)	31 (27.9)	1.000
Repeat endoscopy	18 (25.4)	18 (16.2)	0.181

^a^High-risk endoscopic features; deep ulcers > 2 mm in depth, large ulcers > 1 cm, extensive involvement

Histological evidence of active inflammation was reported in 129 (71%) patients, 49 (38.0%) of whom had ulcerative and 51 (39.5%) had non-ulcerative inflammation endoscopically; the rest were normal. Among patients with active histological inflammation, 75/129 (58.1%) had concurrent chronic features and 15/129 (11.6%) had concurrent microscopic features. Patients with active inflammation had an earlier onset of symptoms (*P* = 0.014) and longer duration of symptoms compared with patients with no active inflammation (*P* = 0.102; Table 5). Interestingly, more patients with active inflammation had grade ≥ 3 diarrhea or colitis than did those with no active inflammation (*P* = 0.001 and *P =* 0.012). A higher proportion (48.8%) of patients with active inflammation had high-risk features on endoscopic evaluation (*P* < 0.001). In addition, patients with active inflammation had higher rates of symptom recurrence (*P* = 0.004) and repeat endoscopy (*P* = 0.007).

**Table 5:** Association between histological active inflammation and clinical characteristics

Characteristic	Active inflammation *N* = 129	No-active inflammation *N* = 53	*P* value
Time from ICPI to onset (months, SD)	3 (4)	5 (9)	0.014
Duration of symptoms (days, SD)	39 (96)	17 (21)	0.102
Diarrhea grade, n (%)			0.001
1	9 (7.0)	15 (28.3)	
2	43 (33.3)	17 (32.1)	
3–4	77 (59.7)	21 (39.6)	
Colitis grade, n (%)			0.012
1	19 (14.7)	18 (34.0)	
2	63 (48.8)	23 (43.4)	
3–4	47 (36.4)	12 (22.6)	
IV steroids, n (%)	71 (54.0)	13 (24.5)	< 0.001
Duration of steroid (days, SD)	67 (77)	37 (34)	0.083
Infliximab/vedolizumab, n (%)	39 (33.6)	3 (12.0)	0.032
Colonoscopy findings, n (%)			< 0.001
Ulcer	49 (38.0)	0 (0.0)	
Non-ulcerative inflammation	51 (39.5)	15 (28.3)	
Normal	29 (22.5)	38 (71.7)	
High-risk endoscopic features, n (%)	63 (48.8)	8 (15.1)	< 0.001
Outcomes, n (%)			
Hospitalization	98 (76.0)	34 (64.2)	0.143
Duration of hospitalization (days, SD)	8 (6)	6 (8)	0.196
ICU admission	2 (1.6)	5 (9.4)	0.023
Recurrence	44 (34.1)	7 (13.2)	0.004
Repeat endoscopy	32 (24.8)	4 (7.5)	0.007

Abbreviation: *ICPI* immune checkpoint inhibitor, *ICU* intensive care unit, *IV* intravenous, *SD* standard deviation

#### Clinical outcomes

One hundred thirty-two (72.5%) patients were hospitalized. Colonic perforation occurred in 4 (2.2%) patients. Two underwent conservative treatment with antimicrobial agents and intravenous fluids. One patient developed sepsis and was admitted to the ICU to receive hemodynamic support, in addition to antimicrobial agents. The last patient required an emergent colectomy.

A total of 51 (28.0%) patients experienced recurrent symptoms; 23 had received only corticosteroid therapy for the initial episode and 12 had needed infliximab or vedolizumab in addition to steroids, the rest were treated symptomatically. ICPI treatment was permanently discontinued in 135 (74.2%) patients, and temporarily halted in 47 (25.8%), of whom, 22/47 (46.8%) had recurrent diarrhea.

Overall, 36 (19.8%) patients underwent repeat endoscopic evaluation with a mean follow-up duration of 6 months. Among the 12 patients with evidence of mucosal ulceration on the initial endoscopy, 3 had persistent ulcerations and 9 had healed ulcers to non-ulcerative inflammation. Kaplan-Meier survival analysis revealed that patients with active histological inflammation had comparable OS duration to patients with no active inflammation on histology (*P* = 0.1087; Additional file 1: Figure S5). Likewise, high-risk endoscopic features were not associated with better OS rates (*P* = 0.7377; Additional file 1: Figure S6). Patients who had severe IDC that required immunosuppression had similar survival rates to those who had milder IDC (*P* = 0.2914; Additional file 1: Figure S7). Overall survival duration of patients who had grade 1–2 diarrhea was comparable to that of patients with grade 3–4 (*P* = 0.7965; Additional file 1: Figure S8).

In our cohort, all patients except 5 (97.3%) achieved clinical remission with complete tapering of steroids for the first IDC episode. Amongst patients who had abnormal endoscopy findings initially and underwent repeat endoscopy after IDC treatment, 23/27 (85%) had endoscopic remission. By contrast, histological remission was achieved in 9 (30%) out of the 30 patients who had active histology features initially and had repeat endoscopy with biopsy. Four out of the 34 patients with active inflammation initially and underwent repeat endoscopy did not have biopsy.

#### Factors associated with outcomes

On univariate logistic regression, active histological inflammation, resumption of ICPI treatment, and longer duration of steroid treatment were associated with recurrent diarrhea (*P* < 0.01 for all). On multivariate analysis, no significant associations were sustained between recurrent diarrhea and clinical characteristics (Additional file 1: Tables S6 and S7). On univariate logistic regression, high-risk endoscopic features, active histological inflammation, CTLA-4 based therapy, and longer duration of ICPI treatment were associated with higher requirement for add-on infliximab/vedolizumab therapy (*P* < 0.05 for all; Additional file 1: Table S8). On multivariate analysis, only high-risk endoscopic features were associated with higher requirement for infliximab/vedolizumab therapy (*P* < 0.01; Table 6). On univariate logistic regression, high-risk endoscopic features, CTLA-4 based therapy, and longer duration of ICPI treatment were associated with higher probability of hospital admissions (*P* < 0.05 for all; Additional file 1: Table S8). On multivariate analysis, only the duration of ICPI treatment was associated with higher requirement for admissions (*P* < 0.01; Table 6).

**Table 6:** Multivariate logistic regression analysis of infliximab/vedolizumab use and hospital admission

Characteristic	Infliximab/vedolizumab use		Hospital admission	
	OR (95% CI)	*P* value	OR (95% CI)	*P* value
Age	0.98 (0.95–1.01)	0.20	1.00 (0.98–1.02)	0.93
CTLA-4 based therapy	1.92 (0.68–5.37)	0.22	1.26 (0.58–2.74)	0.55
Duration of ICPI treatment	1.00 (0.99–1.01)	0.17	1.00 (1.00–1.01)	< 0.01
High-risk endoscopic features	3.93 (1.69–9.12)	< 0.01	1.74 (0.79–3.87)	0.17
Active histological inflammation	2.32 (0.60–8.99)	0.22	1.39 (0.64–3.02)	0.40

Abbreviation: *ICPI* immune checkpoint inhibitor, *CTLA-4* cytotoxic T-lymphocyte antigen-4, *OR* odds ratio, *CI* confidence interval

## Discussion

This retrospective study sheds light on the importance of the endoscopic and histological characteristics of IDC and their associations with disease outcomes. We stratified endoscopic findings as high- and low-risk in terms of steroid treatment responsiveness, likewise, histological features as active or no active inflammation. This strategy differs from those documented in previously published studies [15, 16]. The rationale for this strategy stems from the endoscopic and histological overlap between IDC and IBD. It is imperative to identify endoscopic and histological factors that are associated with disease outcomes in a timely manner to pave the way for appropriate treatment recommendations.

Among one of the most notable findings of this study is that the timely performance of endoscopic evaluation (≤ 7 days) decreased the overall duration of symptoms, steroid treatment, as well as hospitalization. Remarkably, the delay in performing endoscopic evaluation (> 30 days) until failure of the first steroid tapering trial led to a delay in initiating guided management, evident by the longer duration from IDC onset to the first infliximab/vedolizumab dose. Although, there was no difference between the two groups in the overall requirement for infliximab/vedolizumab add-on therapy. Subsequently, this resulted in more recurrence and longer duration of steroids. Additionally, the delay in the initiation of infliximab/vedolizumab in the group that had delayed endoscopic evaluation with a resulting inadequate treatment of IDC could explain the finding of a similar proportion of endoscopic and histological findings among the two groups.

Interestingly, patients with active histological inflammation had earlier time to symptom onset, longer duration of symptoms, and higher disease severity. In addition, this correlation was found to have treatment implications, as patients with active histological inflammation required more intravenous corticosteroids and infliximab/vedolizumab infusions due to steroid-refractory disease. The diagnostic correlation between active inflammation and endoscopic features is also worthy of mention, where this trend was also evident in patients with high-risk endoscopic features by the need for more infliximab/vedolizumab infusions. This finding is concordant with that of a previously published series of 92 patients who had developed IDC [11]. In contrast, patients who had immune-mediated diarrhea with normal endoscopy and histology had milder disease course and less recurrence of symptoms.

As the need for immunosuppressive treatment in patients with grade 3 and 4 IDC is obvious, contrariwise to patients with grade 1 IDC, we assessed the value of endoscopy and histology in patients with grade 2 IDC separately, where there is uncertainty about immunosuppression use. Prodigiously, timely endoscopy to guide the early introduction of steroids as well as infliximab or vedolizumab was associated with shorter duration of IDC symptoms and lower recurrence rate. To confirm our observation, the presence of high-risk endoscopic features and active histological inflammation was more frequent in patients who received immunosuppression.

The utility of laboratory studies is worthy of mention as well. Fecal calprotectin and lactoferrin have been established as useful, cost effective, and non-invasive methods of identifying active inflammation [17]. In our study, calprotectin levels and qualitative lactoferrin correlated with endoscopic and histological findings. Lactoferrin was comparatively more sensitive at detecting histological than endoscopic inflammation. These non-invasive tests provide valuable information about the overall disease status and should be used in practice, particularly before a diagnostic endoscopy, to delineate their value as initial screening tests for the presence of ulcerative inflammation. Calprotectin may be considered for follow-up of disease activity according to recent American Society of Clinical Oncology recommendations [14].

There is substantial debate on the optimal surrogate marker for identifying disease remission in IBD field; it varies from resolution of symptoms to no evidence of disease on endoscopy. It was recently suggested that histological remission is a better surrogate marker of disease remission since patients who do not show any endoscopic evidence of disease tend to have persistent symptomatology and histological inflammation [18]. However, the optimal target on histological or endoscopic remission has not been established.

Although the clinically significant histological characterization of IDC adds to our understanding of this disease entity, achieving histological remission in practice could prove to be cumbersome, requiring longer therapy and follow-up surveillance, which may not be very cost effective. Hence, characterizing endoscopic features, such as the presence of high-risk features that are correlated with a histological profile that has clinical implications, may be an appropriate strategy in the management of IDC. In addition, since endoscopic characterization is correlated with the need for additional immunosuppressant treatment, early diagnostic endoscopic findings should be used to guide treatment strategies, particularly those concerning the need for additional therapy. Focus should also be diverted towards achieving endoscopic remission rather than histological remission, since there is a favorable overlap between the two entities.

The above-mentioned findings lend support to the use of a personalized treatment strategy for IDC. An index diagnostic endoscopy is crucial for characterizing disease features, even if a clinical suspicion of IDC is deemed sufficient to initiate treatment. In our cohort, ~ 10% of patients had isolated right colon/terminal ileum involvement; therefore, full extent colonoscopy should be the preferred procedure to establish IDC diagnosis. Subsequently, according to the location on the first colonoscopy, the type of follow-up procedure can be determined using either flexible sigmoidoscopy or colonoscopy. The knowledge that a patient has active histological inflammation or high-risk endoscopic features should be used to guide timely management decisions, particularly those concerning the initiation of add-on drug such as infliximab or vedolizumab. In addition, it is crucial to identify patients who are at risk of perforation and determine whether early add-on therapy can prevent such serious adverse events.

Only a few studies have been performed on the efficacy of the early introduction of immunosuppressive treatment in the setting of IDC [19, 20]; however, there is ample evidence to support the early addition of an immunosuppression strategy involving infliximab and azathioprine in the setting of IBD, with studies showing higher rates of mucosal healing compared to conventional therapy or a placebo combination [21, 22]. Owing to the similarities in the histological and endoscopic findings of IDC and IBD, such an approach may prove to be efficacious in preventing long disease course, long duration of steroid treatment, symptom recurrence, and subsequent re-hospitalization. We previously reported a trend of a lower duration of corticosteroid therapy in patients who received infliximab therapy compared to those who did not [23]. Future studies should assess whether early initiation of infliximab or vedolizumab can affect the duration of steroid therapy and result in early disease remission.

In addition to the findings mentioned above, the effect of active inflammation on patient outcomes is noteworthy, particularly the statistically significant associations between active inflammation with symptom recurrence and repeat endoscopy. Patients’ perception of outcomes is comparatively short term and is mostly focused on symptom improvement. Hence, recurring symptoms can have an adverse effect on patients’ quality of life, which is an extremely important endpoint to consider in cancer patients. In addition, recurring symptoms can prompt re-hospitalization, and intuitively, they would pave the way for a battery of in-hospital costs, which could prove to be burdensome for the patient as well as the healthcare system. Preventing re-hospitalization in patients with IDC would reduce healthcare costs, particularly since the indications for ICPI are expected to increase in the future. Last, whether endoscopic remission could be used as a surrogate marker that could prompt the resumption of ICPI therapy should be investigated in future studies.

Our study sheds light on multiple clinically significant associations that need to be further validated in future studies. Nonetheless, there are notable limitations to our study. Besides the inherent drawbacks of a retrospective design, our study may have been underpowered in certain subgroups, especially the repeat endoscopy subgroup, limiting our findings. In addition, the limited number of events in our study, particularly colonic perforation and ICU admissions, precluded further analysis. Our cohort could be biased by the fact that usually not all patients undergo endoscopic evaluation, only those with more severe disease. In patients who only underwent flexible sigmoidoscopy, extensive disease or pan colitis could not be ruled out and hence represents a source of potential bias. Last, the treatment strategies used for the management of GI-irAEs were not standardized and were based on the clinical judgement of the treating physician.

In conclusion, endoscopic evidence of high-risk features or presence of active inflammation on histological examination represent important markers of disease severity with clinical implications and should be used to devise GI-irAE-focused treatment algorithms that incorporate a more intricate degree of specificity to improve upon the currently available guidelines. In patients with histological evidence of active inflammation or endoscopic evidence of high-risk features, early initiation of add-on therapy should be given serious consideration to avoid symptom recurrence as well as re-hospitalization, thereby maintaining patients’ quality of life and improving patient outcomes. Fecal calprotectin and lactoferrin assays should be used early in the disease course to establish a trend and delineate the utility of the tests in monitoring disease. Further prospective studies are needed to define the appropriate timing for early as well as conventional combination immunosuppressive therapy. In addition, the utility of resolving active histological inflammation or high-risk endoscopic features requires further validation.

## Additional file

Additional file 1: **Figure S1**. Incidence of colitis. **Figure S2**. Flow chart of endoscopic findings, histologic features, and immunosuppressive treatment. **Figure S3**. Endoscopy images demonstrating: (a) high-risk features, (b) low-risk features, (c) Ulcerative colitis like disease, (d) Crohn’s like disease; yellow arrow demonstrates large deep mucosal ulceration surrounded by normal mucosa. **Figure S4**. Histopathology images demonstrating: (a) colonic mucosa with architecture distortion, basal plasmacytosis (white arrow), cryptitis (yellow arrow) and crypt abscess (red arrow), (b) Colonic mucosa with mild architecture distortion and minimal evidence of active inflammation. **Figure S5**. Kaplan-Meier curve showing comparable overall survival between patients with active histological inflammation and those with no active inflammation (*P* = 0.1087). **Figure S6**. Kaplan-Meier curve showing comparable overall survival between patients with high-risk endoscopic features and those without (*P* = 0.7377). **Figure S7**. Kaplan-Meier curve showing comparable overall survival rates between patients who received immunosuppression for IDC and those who did not (*P* = 0.2914). **Figure S8**. Kaplan-Meier curve showing comparable overall survival between patients who had grade 1–2 and those who had grade 3–4 diarrhea (*P* = 0.7965). (DOCX 5087 kb)

## Abbreviations

CI: Confidence interval
CT: Computed tomography
CTLA-4: Cytotoxic T-lymphocyte-associated protein 4
GI: Gastrointestinal
IBD: Inflammatory bowel disease
ICPI: Immune checkpoint inhibitor
IDC: Immune-mediated diarrhea and colitis
IrAE: Immune-related adverse event
OR: Odds ratio
PD-1: Programmed cell death protein-1
PD-L1: Programmed death-ligand 1
SD: Standard deviation
