# Supplementary material for: Importance of endoscopic and histological evaluation in the management of immune checkpoint inhibitor-induced colitis
Source: J Immunother Cancer. 2018 Sep 25;6:95. doi: 10.1186/s40425-018-0411-1 (PMC6156850; doi:10.1186/s40425-018-0411-1)
Supplement: Supplementary file 1 — Figure S1. Incidence of colitis. Figure S2. Flow chart of endoscopic findings, histologic features, and immunosuppressive treatment. Figure S3. Endoscopy images demonstrating: (a) high-risk features, (b) low-risk features, (c) Ulcerative colitis like disease, (d) Crohn’s like disease; yellow arrow demonstrates large deep mucosal ulceration surrounded by normal mucosa. Figure S4. Histopathology images demonstrating: (a) colonic mucosa with architecture distortion, basal plasmacytosis (white arrow), cryptitis (yellow arrow) and crypt abscess (red arrow), (b) Colonic mucosa with mild architecture distortion and minimal evidence of active inflammation. Figure S5. Kaplan-Meier curve showing comparable overall survival between patients with active histological inflammation and those with no active inflammation (P = 0.1087). Figure S6. Kaplan-Meier curve showing comparable overall survival between patients with high-risk endoscopic features and those without (P = 0.7377). Figure S7. Kaplan-Meier curve showing comparable overall survival rates between patients who received immunosuppression for IDC and those who did not (P = 0.2914). Figure S8. Kaplan-Meier curve showing comparable overall survival between patients who had grade 1–2 and those who had grade 3–4 diarrhea (P = 0.7965). (DOCX 5087 kb) [file 40425_2018_411_MOESM1_ESM.docx]

**Additional file 1: Table S1**. Association between clinical characteristics and endoscopic findings

| Characteristic | Ulcers  N = 49 | Non-ulcer inflammation  N = 66 | Normal  N = 67 | P value |
| --- | --- | --- | --- | --- |
| Checkpoint inhibitor type, n (%) |  |  |  | < 0.001 |
| CTLA-4 | 30 (61.2) | 27 (40.9) | 14 (20.9) |  |
| PD-1/L-1 | 10 (20.4) | 22 (33.3) | 35 (52.2) |  |
| Combination^b^ | 9 (18.4) | 17 (25.8) | 18 (26.9) |  |
| Time from ICPI to onset (SD) | 75 (96) | 89 (100) | 161 (258) | 0.136 |
| Colitis treatment, n (%) |  |  |  | < 0.001 |
| Immunosuppressant | 44 (89.8) | 57 (86.4) | 40 (59.7) |  |
| Non-immunosuppressant | 5 (10.2) | 9 (13.6) | 27 (40.3) |  |
| Duration of steroid (days, SD) | 70 (101) | 58 (45) | 60 (71) | 0.639 |
| Mesalamine, n (%) | 12 (24.5) | 20 (30.3) | 15 (22.4) | 0.565 |
| Diarrhea grade, n (%) |  |  |  | 0.007 |
| 1 | 5 (10.2) | 5 (7.6) | 14 (20.9) |  |
| 2 | 16 (32.7) | 16 (24.2) | 28 (41.8) |  |
| 3-4 | 28 (57.1) | 45 (68.2) | 25 (37.3) |  |
| Colitis grade, n (%) |  |  |  | < 0.001 |
| 1 | 2 (4.1) | 10 (15.2) | 25 (37.3) |  |
| 2 | 23 (46.9) | 29 (43.9) | 34 (50.7) |  |
| 3-4 | 24 (49.0) | 27 (40.9) | 8 (11.9) |  |
| Outcomes, n (%) |  |  |  |  |
| Hospitalization | 39 (79.6) | 56 (84.8) | 37 (55.2) | < 0.001 |
| ICU | 1 (2.0) | 4 (6.1) | 2 (3.0) | 0.473 |
| Recurrent | 14 (28.6) | 17 (25.8) | 20 (29.9) | 0.867 |
| Repeat endoscopy | 12 (24.5) | 15 (22.7) | 9 (13.4) | 0.253 |
| Mean follow-up duration (months, SD) | 3 (2) | 6 (7) | 8 (9) | 0.111 |
| Repeat endoscopic findings^c^, n (%) |  |  |  | < 0.001 |
| Ulcer | 3 (25.0) | 0 (0.0) | 0 (0.0) |  |
| Non-ulcerative inflammation | 9 (75.0) | 8 (50.0) | 1 (11.1) |  |
| Normal | 0 (0.0) | 8 (50.0) | 8 (88.9) |  |

Abbreviation: ICPI, immune checkpoint inhibitor; CTLA-4, cytotoxic T-lymphocyte antigen-4; ICU, intensive care unit; SD, standard deviation; PD-1/L-1, programmed cell death receptor-1 and ligand 1.

^a^ Includes non-ulcerative endoscopic inflammation cases (*n* = 22) and normal endoscopies with positive histological inflammation (*n* = 7).

^b^ Combination: CTLA-4 and PD-1/L-1

^c^ Only patients who underwent repeat endoscopy

**Additional file 1: Table S4.** Lactoferrin sensitivity

| **Evaluation** | **Positive** | **Negative** |
| --- | --- | --- |
| Endoscopic findings |  |  |
| Abnormal | 42 (70) | 4 (36) |
| Normal | 18 (30) | 7 (64) |
| Histological findings |  |  |
| Abnormal | 54 (90) | 8 (73) |
| Normal | 6 (10) | 3 (27) |

1. Sensitivity of lactoferrin to detect endoscopic inflammation = 70%
2. Sensitivity of lactoferrin to detect histological inflammation = 90%

**Additional file 1: Table S3.** Characteristics of patients with grade 2 diarrhea (n = 60) by timing of endoscopy.

| Characteristic | > 7 days after diarrhea onset  N = 28 | ≤ 7 days after diarrhea onset  N = 32 | *P* value |
| --- | --- | --- | --- |
| IV steroids, n (%) | 7 (41.2) | 5 (22.7) | 0.299 |
| Duration of symptoms (days, SD) | 63 (110) | 12 (14) | 0.025 |
| Duration of steroid (days, SD) | 85 (90) | 44 (39) | 0.077 |
| Infliximab/vedolizumab, n (%) | 6 (21.4) | 7 (21.9) | 1.000 |
| Duration from onset to first infliximab/vedolizumab dose (days, SD) | 25 (22) | 5 (3) | 0.087 |
| Colonoscopy findings, n (%) |  |  | 0.130 |
| Ulcer | 8 (28.6) | 8 (25.0) |  |
| Non-ulcerative inflammation | 4 (14.3) | 12 (37.5) |  |
| Normal | 16 (57.1) | 12 (37.5) |  |
| High-risk endoscopic features, n (%) | 7 (25.0) | 12 (37.5) | 0.406 |
| Active histological inflammation, n (%) | 20 (71.4) | 23 (71.9) | 1.000 |
| Outcomes, n (%) |  |  |  |
| Hospitalization | 12 (42.9) | 25 (78.1) | 0.008 |
| Duration of hospitalization (days, SD) | 9 (5) | 5 (2) | < 0.001 |
| ICU admission | 0 (0.0) | 1 (3.1) | 1.000 |
| Recurrence | 12 (42.9) | 5 (15.6) | 0.024 |

**Additional file 1:** **Table S4.** Characteristics of patients with grade 2 diarrhea (n = 60) by colitis treatment.

| Characteristic | Immunosuppression  N = 43 | No-immunosuppression  N = 17 | *P* value |
| --- | --- | --- | --- |
| Duration of symptoms (days, SD) | 50 (97) | 10 (10) | 0.100 |
| Colonoscopy findings, n (%) |  |  | 0.050 |
| Ulcer | 15 (34.9) | 1 (5.9) |  |
| Non-ulcerative inflammation | 11 (25.6) | 5 (29.4) |  |
| Normal | 17 (39.5) | 11 (64.7) |  |
| High-risk endoscopic features, n (%) | 17 (39.5) | 2 (11.8) | 0.063 |
| Active histological inflammation, n (%) | 38 (88.4) | 5 (29.4) | < 0.001 |
| Outcomes, n (%) |  |  |  |
| Hospitalization | 27 (62.8) | 10 (58.8) | 0.777 |
| Duration of hospitalization (days, SD) | 7 (4) | 4 (3) | 0.113 |
| ICU admission | 1 (2.3) | 0 (0.0) | 1.000 |
| Recurrence | 16 (37.2) | 1 (5.9) | 0.024 |
| Repeat endoscopy | 11 (25.6) | 1 (5.9) | 0.151 |

**Additional file 1: Table S5.** Association between clinical characteristics and endoscopic findings

| Characteristic | Normal endoscopy and histology  N = 28 | Abnormal endoscopy or histology  N = 154 | P value |
| --- | --- | --- | --- |
| Duration of symptoms (days, SD) | 15 (20) | 36 (88) | 0.209 |
| Colitis treatment, n (%) |  |  | < 0.001 |
| Immunosuppressant | 11 (39.3) | 130 (84.4) |  |
| Non-immunosuppressant | 17 (60.7) | 24 (15.6) |  |
| Duration of steroid (days, SD) | 34 (26) | 65 (75) | 0.168 |
| Infliximab/vedolizumab, n (%) | 0 (0.0) | 42 (32.3) | 0.034 |
| Diarrhea grade, n (%) |  |  | 0.010 |
| 1 | 9 (32.1) | 15 (9.7) |  |
| 2 | 8 (28.6) | 52 (33.8) |  |
| 3-4 | 11 (39.3) | 87 (56.5) |  |
| Outcomes, n (%) |  |  |  |
| Hospitalization | 15 (53.6) | 117 (76.0) | 0.021 |
| ICU | 1 (3.6) | 6 (3.9) | 1.000 |
| Resumed ICPI | 11 (39.3) | 37 (24.0) | 0.105 |
| Recurrent overall | 3 (10.7) | 48 (31.2) | 0.037 |
| After ICPI resumption^a^ | 3 (27.3) | 19 (51.4) | 0.189 |
| Repeat endoscopy | 2 (7.1) | 34 (22.1) | 0.075 |

Abbreviation: ICPI, immune checkpoint inhibitor; ICU, intensive care unit; SD, standard deviation.

^a^The percentages and *P* value were calculated for only patients who resumed ICPI treatment.

**Additional file 1: Table S6.** Univariate logistic regression analysis of recurrent diarrhea

| Characteristic | OR (95% CI) | *P* value |
| --- | --- | --- |
| Age | 0.99 (0.98–1.02) | 0.66 |
| CTLA-4 based therapy | 1.78 (0.73–4.35) | 0.35 |
| Duration of ICPI treatment | 1.01 (1.00–1.01) | 0.18 |
| Resumption of ICPI treatment | 3.06 (1.52–6.18) | < 0.01 |
| High-risk endoscopic features | 1.01 (0.52–1.96) | 0.97 |
| Active histological inflammation | 3.40 (1.42–8.17) | < 0.01 |
| Steroid treatment duration | 1.01 (1.00–1.02) | 0.05 |
| Infliximab/vedolizumab use | 1.20 (0.57–2.55) | 0.63 |
| Duration from onset to infliximab/vedolizumab | 1.04 (0.99–1.09) | 0.07 |
| Duration from onset to endoscopic evaluation | 1.02 (1.01–1.03) | < 0.01 |
| Abbreviation: ICPI, immune checkpoint inhibitor; CTLA-4, cytotoxic T-lymphocyte antigen-4; OR, odds ratio; CI, confidence interval. | | |

**Additional file 1: Table S7.** Multivariate logistic regression analysis of recurrent diarrhea

| Characteristic^a^ | OR (95% CI) | *P* value |
| --- | --- | --- |
| Resumption of ICPI treatment | 5.97 (0.73–48.74) | 0.09 |
| Active histological inflammation | 4.08 (0.11–148.71) | 0.44 |
| Steroid treatment duration | 1.04 (1.00–1.07) | 0.05 |
| Duration from onset to infliximab/vedolizumab | 1.01 (0.95–1.07) | 0.81 |
| Duration from onset to endoscopic evaluation | 1.01 (0.96–1.06) | 0.73 |
| Abbreviation: ICPI, immune checkpoint inhibitor; OR, odds ratio; CI, confidence interval.  ^a^Factors with *P* < 0.1 in the univariate analysis were included in the multivariate analysis. | | |

**Additional file 1: Table S8.** Univariate logistic regression analysis of infliximab/vedolizumab use and hospital admission

| Characteristic | Infliximab/vedolizumab use | | Hospital admission | |
| --- | --- | --- | --- | --- |
|  | **OR (95% CI)** | ***P* value** | **OR (95% CI)** | ***P* value** |
| Age | 0.99 (0.96–1.01) | 0.27 | 1.00 (0.98–1.02) | 0.92 |
| CTLA-4 based therapy | 3.12 (1.26–7.72) | 0.01 | 2.14 (1.10–4.16) | 0.03 |
| Duration of ICPI treatment | 1.01 (1.00–1.01) | 0.04 | 1.00 (1.00–1.01) | < 0.01 |
| High-risk endoscopic features | 4.57 (2.08–10.03) | < 0.01 | 2.23 (1.09–4.58) | 0.03 |
| Active histological inflammation | 4.39 (1.05–13.18) | 0.04 | 1.77 (0.89–3.53) | 0.11 |
|  | | | | |

Abbreviation: ICPI, immune checkpoint inhibitor; CTLA-4, cytotoxic T-lymphocyte antigen-4; OR, odds ratio; CI, confidence interval.

**Additional file 1: Figure S1.** Incidence of colitis

PD-1/L1 3038

CTLA-4 1826

Any grade IDC

197 (6.5%)

Combination 380

Monotherapy 1446

Endoscopy 67 (2.2%)

Endoscopy 71 (4.9%)

Endoscopy 44 (11.6%)

Any grade IDC

147 (10.2%)

Any grade IDC

93 (24.5%)

**Additional file 1: Figure S2**. Flow chart of endoscopic findings, histologic features, and immunosuppressive treatment.

Histology

No high-risk features

111 (61%)

Steroids: 19 (42%)

IFX/vedo: 3 (7%)

Steroids: 57 (86%)

IFX/vedo: 18 (27%)

Steroids: 6 (75%)

IFX/vedo: 1 (13%)

Steroids: 60 (95%)

IFX/vedo: 31 (49%)

Active

63 (89%)

Inactive

8 (11%)

Inactive

45 (41%)

Active

66 (59%)

High-risk features

71 (39%)

**Additional file 1: Figure S3.** Endoscopy images demonstrating: (a) high-risk features, (b) low-risk features, (c) Ulcerative colitis like disease, (d) Crohn’s like disease; yellow arrow demonstrates large deep mucosal ulceration surrounded by normal mucosa.


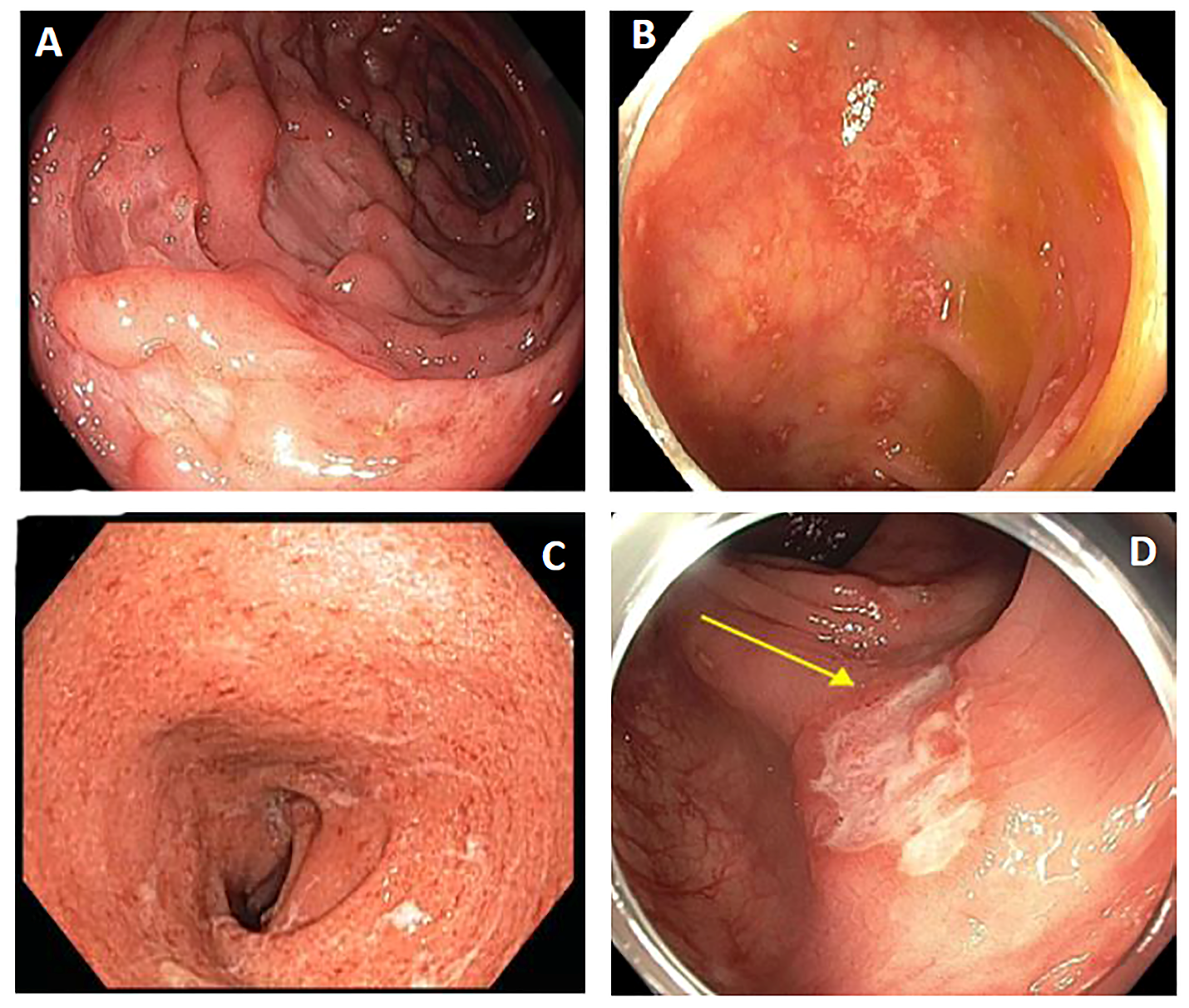


**Additional file 1: Figure S4.** Histopathology images demonstrating: (a) colonic mucosa with architecture distortion, basal plasmacytosis (white arrow), cryptitis (yellow arrow) and crypt abscess (red arrow), (b) Colonic mucosa with mild architecture distortion and minimal features of active inflammation.

**
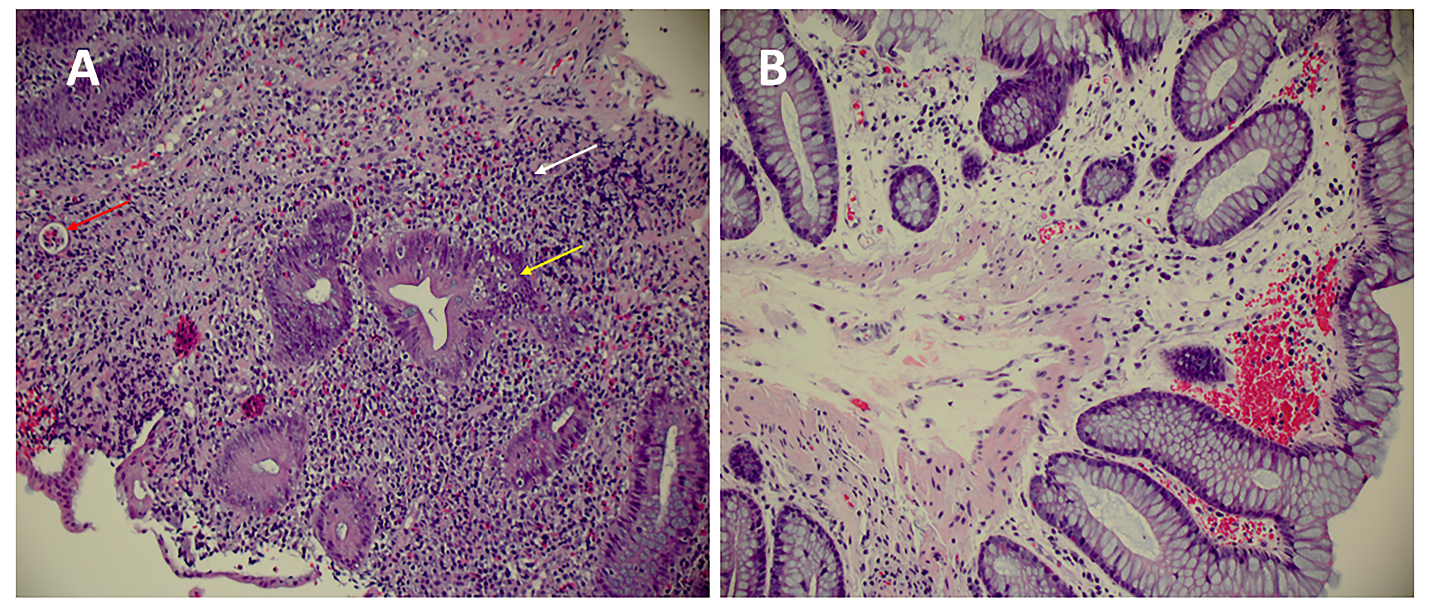
**

**Additional file 1: Figure S5.** Kaplan-Meier curve of overall survival of patients with active histological inflammation.

**Additional file 1: Figure S6.** Kaplan-Meier curve of overall survival of patients with high-risk endoscopic features.

**Additional file 1: Figure S7.** Kaplan-Meier curve of overall survival of patients received immunosuppression for IDC.

**Additional file 1: Figure S8.** Kaplan-Meier curve of overall survival by the grade of diarrhea.
